# Diagnostic Performance of Ultrasound-Guided Attenuation Parameter (UGAP) for Hepatic Steatosis Assessment: Comparison with MRI-PDFF and Evaluation of Cohort-Derived Thresholds

**DOI:** 10.3390/diagnostics16132006

**Published:** 2026-06-27

**Authors:** Dimitrios Kavvadas, Natalia-Valeria Pentara, Dimitrios Kourdakis, Aris Liakos, Emmanouil Sinakos, Panos Prassopoulos, Vasileios Rafailidis

**Affiliations:** 1 Department of Clinical Radiology, AHEPA University Hospital, Aristotle University of Thessaloniki, 54636 Thessaloniki, Greece; navapent@gmail.com (N.-V.P.); dimitriskourd1@gmail.com (D.K.); pprasopo@auth.gr (P.P.); vrafaili@auth.gr (V.R.); 2 Clinical Research and Evidence-Based Medicine Unit, Second Medical Department, Aristotle University of Thessaloniki, 54642 Thessaloniki, Greece; arliakos@gmail.com; 3 Fourth Medical Department, Aristotle University of Thessaloniki, 54642 Thessaloniki, Greece; em_sinakos@yahoo.com

**Keywords:** hepatic steatosis, UGAP, MRI-PDFF, MASLD, ultrasound, liver fat quantification

## Abstract

**Background/Objectives**: To evaluate the diagnostic performance of ultrasound-guided attenuation parameter (UGAP) for the assessment of hepatic steatosis in a population at risk for metabolic dysfunction-associated steatotic liver disease (MASLD), using MRI proton density fat fraction (PDFF) as the reference standard, and to also derive optimal population-specific diagnostic thresholds. **Methods**: In this single-center prospective study, 64 adults at risk for MASLD underwent UGAP measurement and MRI-PDFF. UGAP was performed according to standardized manufacturer-recommended protocols and standardized on the right hepatic lobe. Hepatic steatosis was staged using established MRI-PDFF thresholds. Diagnostic performance was evaluated using receiver operating characteristic (ROC) analysis. Cohort-UGAP cut-offs were derived using the Youden index. Associations between UGAP and clinical parameters were assessed using correlation and regression analyses. **Results**: UGAP correlated strongly with MRI-PDFF (ρ = 0.82, *p* < 0.001). The areas under the ROC curve (AUCs) for detecting mild, moderate, and severe steatosis were 0.86, 0.96, and 0.96, respectively. Right-lobe acquisitions outperformed left-lobe measurements, while four-region averaging yielded the highest diagnostic performance. UGAP values were associated with BMI, waist circumference, and liver enzymes. **Conclusions**: UGAP provides an accurate noninvasive assessment of hepatic steatosis, demonstrating high overall diagnostic agreement with MRI-PDFF. Right-lobe acquisition and multi-regional averaging further improve its performance. While cohort-specific threshold optimization may enhance clinical applicability, larger studies are needed to fully confirm its accuracy in advanced stages.

## 1. Introduction

Metabolic dysfunction-associated steatotic liver disease (MASLD) has emerged as the most prevalent chronic liver condition worldwide, closely linked to obesity, insulin resistance, dyslipidemia, and cardiovascular disease [1,2]. MASLD is increasingly recognized as a dominant cause of liver disease progression, leading to the eventual need for liver transplantation [1]. With a worldwide prevalence of 38% and up to 70% in obese individuals and those with type 2 diabetes mellitus [1,3], early and accurate detection of hepatic steatosis is critical for disease monitoring and timely therapeutic interventions [4].

Magnetic resonance imaging (MRI) proton density fat fraction (PDFF) is regarded as the noninvasive reference standard for quantitative liver fat assessment [5,6]. Meta-analyses have confirmed the high diagnostic accuracy and reproducibility of MRI-PDFF for grading hepatic steatosis (AUC: 0.90–0.98), leading to its widespread clinical use [7]. However, MRI is limited by high costs, restricted availability, and long examination times, which preclude its use as a population screening or longitudinal monitoring tool [6]. Therefore, ultrasound-based techniques have gathered increasing attention as accessible, noninvasive alternatives for hepatic steatosis assessment, specifically when coupled with fibrosis quantification via elastography [8].

Among these, the ultrasound-guided attenuation parameter (UGAP) quantifies ultrasound beam attenuation within the liver and provides a numerical estimate expressed in dB/cm/MHz. Previous studies have demonstrated promising diagnostic performance of UGAP in detecting hepatic steatosis [9,10,11,12]. However, reported cut-off values and diagnostic accuracy vary across studies, reflecting differences in study populations, acquisition protocols, ultrasound platforms, and reference standards [9,10,11,12,13]. Furthermore, attenuation-based measurements may be influenced by patient-related factors such as body mass index (BMI) and tissue composition, raising concerns regarding the generalizability of vendor-recommended thresholds across different populations [13].

Technical factors, such as intrahepatic regional variability may also impact UGAP-based assessment but remain incompletely characterized. For instance, left-lobe measurements are often affected by cardiac motion, gastric gas, and probe angulation, potentially contributing to greater variability in attenuation measurements compared to the right lobe [8,14]. Consequently, additional validation studies using MRI-PDFF as a quantitative reference standard are necessary to better define the clinical role of UGAP across diverse patient cohorts and standardized imaging protocols.

Recent studies have demonstrated promising diagnostic performance of several ultrasound-based attenuation techniques for hepatic steatosis assessment. However, substantial variability in reported thresholds and diagnostic performance persists across studies, reflecting differences in patient populations, acquisition protocols, ultrasound platforms, and reference standards [11,13,15].

The purpose of the present study was to evaluate the diagnostic performance of UGAP for hepatic steatosis assessment in a population at risk for MASLD using MRI-PDFF as the reference standard. In addition, we aimed to derive optimal population-specific UGAP cut-offs and to compare them with vendor-recommended thresholds. Secondary analyses were performed to investigate the influence of clinical factors on UGAP measurements and to explore intrahepatic regional variability across liver segments.

## 2. Materials and Methods

### 2.1. Study Design and Population

This single-center, prospective study evaluated an adult (≥18 years) population at risk for MASLD, predominantly Mediterranean/Caucasian of Greek origin.

Individuals were considered at risk for MASLD based on the presence of at least one of the following:

- overweight (BMI ≥ 25 kg/m^2^) or obesity (BMI ≥ 30 kg/m^2^);
- type 2 diabetes mellitus;
- abnormal liver enzyme levels, defined as ALT > 31 U/L and/or AST > 34 U/L according to local laboratory reference ranges [16,17].

Exclusion criteria were as follows [18,19]:

- viral hepatitis B or C;
- autoimmune hepatitis;
- primary biliary cholangitis;
- primary sclerosing cholangitis;
- Wilson disease;
- Hemochromatosis;
- significant alcohol consumption (defined as >30 g/day in men and >20 g/day in women);
- known hepatic malignancy;
- contraindications to MRI.

All participants underwent UGAP and MRI-PDFF assessments on the same day. The reproducibility of UGAP has been previously established, with published studies reporting high intra- and inter-observer agreement [20].

A sample size estimation was performed based on ROC analysis for the primary endpoint of assessing the diagnostic performance of UGAP against MRI-PDFF. Assuming an expected AUC of 0.85, a null hypothesis AUC of 0.50, a two-sided significance level (α) of 0.05, and 80% statistical power (β = 0.20), a minimum sample size of 60 participants was estimated to be sufficient. Anticipating a small proportion of potentially non-evaluable examinations or incomplete datasets, recruitment reached 64 participants with complete paired data, thereby providing adequate power for the primary analysis.

The study was conducted in accordance with the Declaration of Helsinki and approved by the local institutional review board (*ref.no.161-2025, Medical School Bioethics Committee, AUTh*). Written informed consent was obtained from all participants.

### 2.2. Ultrasound-Guided Attenuation Parameter (UGAP)

UGAP measurements were performed using a LOGIQ E9 ultrasound system (GE Healthcare, Chicago, IL, USA) equipped with a C1-6 convex transducer (1.0–6.0 MHz), following manufacturer-recommended protocols and current WFUMB guidance [21,22].

All examinations were conducted by an experienced radiologist (with ≥10 years in abdominal ultrasound), with patients in the supine position after overnight fasting. Measurements were obtained through the right intercostal approach during a 6-s breath-hold time frame. UGAP values were recorded in dB/cm/MHz. Regions of interest (ROIs) were placed in the right hepatic lobe (segments V to VIII), avoiding large vessels, bile ducts, focal lesions, and areas of acoustic shadowing. For each participant, at least 5 valid measurements were acquired, and the results were expressed as the median value. Measurement reliability was assessed using the interquartile range (IQR), where only measurements with an IQR/median ≤ 30% were considered acceptable.

The primary analysis was based on the standardized right-center hepatic acquisition, as typically performed in routine clinical practice. Additional regional measurements (right caudal, right cephalic, left lobe, and four-region averaging) were predefined as secondary exploratory analyses to investigate regional variability and the potential impact of acquisition site on diagnostic performance.

### 2.3. MRI Acquisition and PDFF Quantification

MRI examinations were performed on a 3-Tesla system (MAGNETOM Vida, Siemens Healthineers, Forchheim, Germany) using a standardized liver imaging protocol [23,24]. A quantitative liver fat assessment was conducted using a multi-echo chemical shift-encoded gradient-echo sequence, enabling PDFF estimation. Acquisition parameters were selected according to the manufacturer’s recommended protocols for quantitative liver fat imaging. PDFF maps were generated automatically by the scanner software. To obtain a whole-liver average PDFF, eight regions of interest (ROIs) were placed within the parenchyma, encompassing one ROI per liver segment.

**Figure 1:**
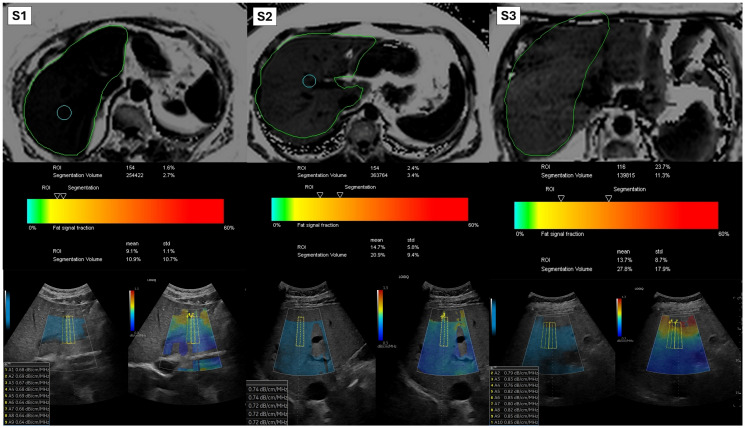
Top row: Axial MRI-PDFF maps demonstrating whole-liver segmentation and region-of-interest placement with increasing hepatic fat fraction across representative cases (S1, S2, and S3 correspond to mild, moderate, and severe hepatic steatosis, respectively). Bottom row: Corresponding UGAP acquisitions obtained from hepatic regions with quantitative attenuation maps and color scales expressed in dB/cm/MHz.

The ROI size ranged from approximately ~2 to 3 cm^2^ to encompass a significant proportion of the liver parenchyma, while avoiding vessels, bile ducts, and artifacts [24,25] (Figure 1).

### 2.4. Definition of Steatosis Stages

Hepatic steatosis was staged based on MRI-PDFF using recently established thresholds [26]:

Mild Steatosis stage-S1: PDFF ≥ 5.75%;

Moderate Steatosis stage-S2: PDFF ≥ 15.5%;

Severe Steatosis stage-S3: PDFF ≥ 21.35%.

For UGAP, the following vendor-recommended cut-offs were used for the initial analysis [21,27]:

S1: 0.65 dB/cm/MHz;

S2: 0.71 dB/cm/MHz;

S3: 0.77 dB/cm/MHz.

### 2.5. Statistical Analysis

For the main analyses and figures, ultrasound measurements were obtained from a standardized central right-lobe region of interest.

Continuous variables were reported as the mean (standard deviation) for MRI-PDFF and the median (interquartile range) for UGAP. Correlations between these metrics and clinical or laboratory parameters were evaluated using Spearman’s rank correlation coefficient.

Diagnostic performance of UGAP in detecting steatosis (S ≥ 1, S ≥ 2, S ≥ 3) was assessed using receiver operating characteristic (ROC) curve analysis by calculating the area under the curve (AUC) and the corresponding 95% confidence intervals. Optimal UGAP cut-offs were derived using the Youden index, and diagnostic performance (sensitivity, specificity, positive predictive value, negative predictive value, and accuracy) was calculated for both optimal and vendor-recommended thresholds. To assess the clinical determinants of UGAP measurements, univariable and multivariable linear regression analyses were performed, including variables such as BMI, waist circumference, and liver enzymes. Exploratory segmental analysis was performed to evaluate variability in diagnostic performance across liver regions.

All statistical tests were two-sided, and a *p*-value < 0.05 was considered statistically significant.

Given the limited number of participants with MRI-PDFF-defined moderate (S2) and severe (S3) steatosis, analyses involving these subgroups were considered exploratory. The resulting ROC-derived thresholds and performance metrics may be less stable than those derived for the overall study population and should therefore be interpreted with appropriate caution.

## 3. Results

### 3.1. Study Population and Steatosis Distribution

In this study, sixty-four participants were included in the final analysis. Baseline demographic, clinical, and imaging characteristics are summarized in Table 1. The mean age was 54.6 ± 15.4 years, and 40 participants (62.5%) were male. The cohort demonstrated a high cardiometabolic burden, with a mean BMI of 32.4 ± 5.7 kg/m^2^, a mean waist circumference of 113.5 ± 14.6 cm, type 2 diabetes mellitus in 36 participants (56.3%), hypertension in 34 (53.1%), and obesity in 34 (53.1%).

The mean whole-liver MRI-PDFF was 10.1 ± 7.9%, while the median UGAP value measured at the right-center hepatic region was 0.71 [IQR: 0.63–0.81] dB/cm/MHz. Based on the MRI-PDFF thresholds, hepatic steatosis (S ≥ 1) was present in 40/64 participants (62.5%). Steatosis stages were distributed as follows: S0 in 24 participants (37.5%), S1 in 26 (40.6%), S2 in 5 (7.8%), and S3 in 9 (14.1%).

### 3.2. Correlations with Demographic and Clinical Parameters

Whole-liver MRI-PDFF demonstrated significant positive correlations with waist circumference (ρ = 0.30, *p* = 0.018), alanine aminotransferase (ALT) (ρ = 0.50, *p* < 0.001), aspartate aminotransferase (AST) (ρ = 0.39, *p* = 0.001), gamma-glutamyl transferase (GGT) (ρ = 0.37, *p* = 0.003), and total cholesterol (ρ = 0.38, *p* = 0.002). Conversely, no significant associations were observed with age, BMI, alkaline phosphatase (ALP), or HbA1c.

UGAP values measured at the right-center hepatic region also correlated significantly with several anthropometric and biochemical parameters, including age (ρ = −0.26, *p* = 0.040), BMI (ρ = 0.29, *p* = 0.019), waist circumference (ρ = 0.39, *p* = 0.001), ALT (ρ = 0.40, *p* = 0.001), AST (ρ = 0.25, *p* = 0.043), GGT (ρ = 0.28, *p* = 0.027), and total cholesterol (ρ = 0.41, *p* < 0.001). No significant correlations were observed with ALP or HbA1c.

### 3.3. Diagnostic Performance and Regional Variations

**Figure 2:**
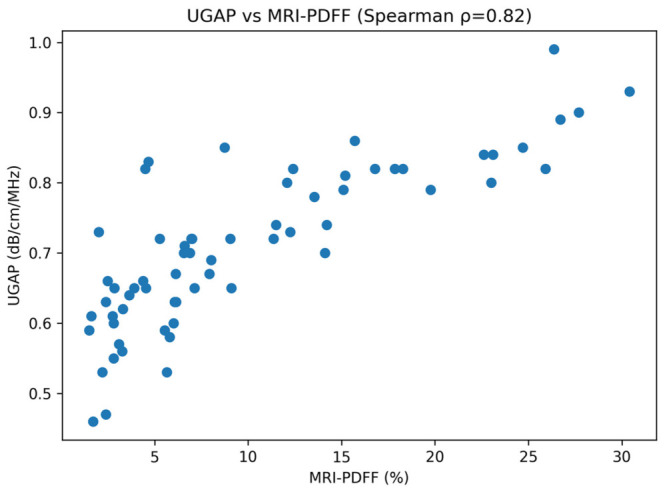
Correlation between right-center UGAP measurements and whole-liver MRI-PDFF values. Each point represents an individual participant. The strong positive correlation (Spearman ρ = 0.82, *p* < 0.001) indicates that increasing ultrasound attenuation measurements are associated with increasing hepatic fat fraction quantified by MRI-PDFF, supporting the validity of UGAP for noninvasive liver fat assessment.

UGAP measurements obtained from the right-center hepatic region demonstrated a strong positive correlation with whole-liver MRI-PDFF (Spearman ρ = 0.82, *p* < 0.001) (Figure 2).

Regional analysis using anatomically matched MRI-PDFF references showed consistently strong correlations across right-lobe acquisition sites. The highest correlation was observed for the right-center region (segments V/VIII; ρ = 0.91, *p* < 0.001), followed by the right-cephalic region (segments VII/VIII; ρ = 0.89, *p* < 0.001) and the right-caudal region (segments V/VI; ρ = 0.86, *p* < 0.001). In contrast, the correlation was lower in the left hepatic lobe (segments II/III; ρ = 0.74, *p* < 0.001) (Table 2).

### 3.4. Diagnostic Performance for Steatosis Staging

**Figure 3:**
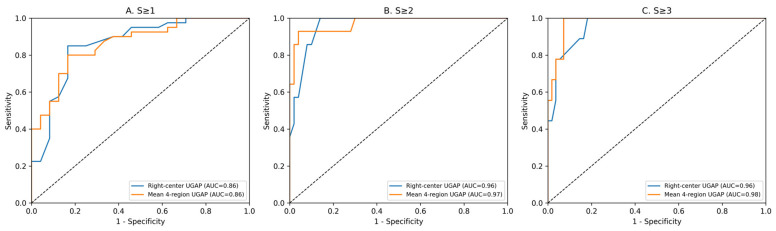
Receiver operating characteristic (ROC) curves demonstrating the diagnostic performance of right-center UGAP measurements for the detection of mild (S ≥ 1), moderate (S ≥ 2), and severe (S ≥ 3) hepatic steatosis using MRI-PDFF as the reference standard. (**A**) shows discrimination for S ≥ 1 steatosis, (**B**) for S ≥ 2 steatosis, and (**C**) for S ≥ 3 steatosis.

ROC analysis demonstrated good to excellent diagnostic performance of right-center UGAP in detecting hepatic steatosis. The AUC values were 0.86 (95% CI: 0.75–0.95) for S ≥ 1, 0.96 (95% CI: 0.91–0.99) for S ≥ 2, and 0.96 (95% CI: 0.91–1.00) for S ≥ 3 (Table 3 and Figure 3).

The optimal Youden-derived thresholds for right-center UGAP were 0.67 dB/cm/MHz for S ≥ 1, 0.79 dB/cm/MHz for S ≥ 2, and 0.80 dB/cm/MHz for S ≥ 3. These thresholds yielded sensitivities of 0.85, 1.00, and 1.00, with corresponding specificities of 0.83, 0.86, and 0.82, respectively.

Notably, multi-regional averaging of UGAP measurements further improved diagnostic performance. Mean four-region UGAP demonstrated AUC values of 0.86 (95% CI: 0.77–0.94) for S ≥ 1, 0.97 (95% CI: 0.92–1.00) for S ≥ 2, and 0.98 (95% CI: 0.94–1.00) for S ≥ 3. Specificity increased notably for advanced steatosis (0.93 for S ≥ 3), while maintaining perfect sensitivity.

To address the limited number of participants in the individual S2 and S3 categories, an additional exploratory ROC analysis was performed using a combined clinically significant steatosis endpoint (MRI-PDFF ≥ 15.5%, corresponding to combined stages S2/S3) (Table 3). Under this clinical endpoint UGAP demonstrated excellent diagnostic performance, with an AUC of 0.96 (95% CI: 0.91–0.99). The Youden-derived threshold was 0.79 dB/cm/MHz, yielding a sensitivity of 1.00 and specificity of 0.86.

A comparison between the manufacturer-recommended and our cohort-derived UGAP thresholds is presented in Table 4. For all steatosis grades, cohort-derived thresholds generally increased specificity and overall diagnostic accuracy, whereas the manufacturer-recommended thresholds favored sensitivity.

To further evaluate factors associated with UGAP measurements, multivariable linear regression analysis was performed including age, BMI, waist circumference, liver enzymes, total cholesterol, and MRI-PDFF. After adjustment for all covariates, MRI-PDFF remained the only significant independent predictor of UGAP values (β = 0.0109, 95% CI: 0.0082–0.0137, *p* < 0.001), whereas age, BMI, waist circumference, liver enzymes, and total cholesterol were not independently associated with UGAP measurements (Table 5). The final model explained 73.7% of the variance in UGAP measurements (R^2^ = 0.737).

## 4. Discussion

In the present study, we evaluated the performance of UGAP for the assessment of hepatic steatosis in an adult Mediterranean/Caucasian population of Greek origin at risk for MASLD, using MRI-PDFF as the reference standard. UGAP demonstrated a strong association with MRI-PDFF, with good diagnostic accuracy for mild steatosis and excellent for moderate to severe steatosis. UGAP measured at the right-center hepatic region showed a strong correlation with whole-liver MRI-PDFF, while segment-matched analyses demonstrated even higher correlations across various right-lobe regions. The strongest association was observed in the right-center region, followed closely by right-cephalic and right-caudal acquisitions, whereas left-lobe measurements showed weaker correlations. These findings support the concept that quantitative attenuation-based ultrasound measurements are influenced not only by hepatic fat content but also by technical and anatomical factors affecting acoustic propagation [28,29]. Prior studies have reported excellent repeatability and interobserver reproducibility for UGAP, supporting its technical robustness when standardized acquisition protocols are applied [20].

In our cohort, ROC analysis demonstrated that UGAP achieved good discrimination for detecting mild steatosis and excellent discrimination for clinically relevant steatosis thresholds (S ≥ 2 and S ≥ 3). Importantly, the Youden-derived thresholds in the present study were slightly higher than the previously suggested vendor cut-offs, particularly for moderate and severe steatosis [27]. For all steatosis grades, cohort-derived thresholds generally increased specificity and overall diagnostic accuracy, whereas manufacturer-recommended thresholds favored sensitivity. The largest difference was observed for moderate steatosis (S ≥ 2), where the cohort-derived threshold of 0.79 dB/cm/MHz increased specificity from 75.6% to 92.7% and overall accuracy from 82.8% to 87.5%, although sensitivity decreased from 95.7% to 78.3%. Similar trends were observed for S ≥ 1 and S3, indicating a clear trade-off between maximizing sensitivity and reducing false-positive classifications. This observation suggests that fixed universal thresholds may not fully capture diagnostic performance across different populations and scanning environments. Cohort-specific factors such as obesity, body habitus, ethnicity, metabolic profile, and hardware/software implementation may influence attenuation-based measurements and should be considered when interpreting quantitative ultrasound thresholds [8,13].

An important practical finding of this study was the effect of the acquisition region. Right-lobe measurements consistently outperformed left-lobe acquisitions in both correlation and classification analyses. This is clinically relevant, as right intercostal access generally provides a longer homogeneous acoustic window with fewer motion artifacts and less interference from adjacent bowel gas. Conversely, left-lobe measurements are often influenced by cardiac motion, gastric gas, reduced depth consistency, and greater variability in probe angulation, all of which contribute to the lower correlations observed in the left lobe [30,31]. Accordingly, the right hepatic lobe should remain the preferred site for UGAP measurements in routine practice.

UGAP also demonstrated significant correlations with anthropometric and metabolic parameters, including waist circumference, BMI, liver enzymes, and total cholesterol. Notably, waist circumference showed stronger associations than BMI, which may reflect the closer relationship between visceral adiposity and hepatic steatosis. These findings further support the metabolic context of attenuation-based liver fat quantification [32]. However, correlations with HbA1c were not significant, suggesting that glycemic control alone may not directly reflect the hepatic fat burden in heterogeneous MASLD populations.

Although attenuation-based ultrasound technologies continue to demonstrate promising diagnostic performance, differences in software implementation, acquisition protocols, and study populations may influence threshold values and reported diagnostic accuracy [33]. Our results support the moderate-to-strong correlations between UGAP and MRI-PDFF and confirm its good diagnostic performance for steatosis detection. The presenting dataset highlights the importance of the acquisition site and the potential benefit of multi-regional averaging. This regional optimization component may represent a clinically relevant refinement for future UGAP and other ultrasound-based fat quantification protocols.

### Limitations

This study has several limitations. First, it was conducted at a single center with a modest sample size, which may limit the external generalizability of our findings. Second, regarding the distribution of steatosis grades, only five participants were classified as S2 and nine as S3 according to MRI-PDFF thresholds. Consequently, ROC-derived thresholds and diagnostic performance estimates for moderate and severe steatosis may be less stable than those for overall steatosis detection. These findings should therefore be considered exploratory and require confirmation in larger cohorts with a more balanced distribution of disease severity. Third, UGAP examinations were performed by a single experienced operator; thus, intra-observer and inter-observer reproducibility were not directly assessed within the present study, although their baseline robustness has been previously well documented. Finally, histologic confirmation was not available. However, MRI-PDFF is widely accepted as the leading noninvasive quantitative reference standard for liver fat assessment and is frequently used in validation studies. Additionally, the study population consisted predominantly of individuals with elevated BMI and cardiometabolic risk, which may influence attenuation thresholds and limit the extrapolation of our results to leaner or ethnically distinct populations.

## 5. Conclusions

Using MRI-PDFF as the reference standard, UGAP demonstrated strong correlation and high diagnostic accuracy for hepatic steatosis assessment in adults at risk for MASLD. Right-lobe acquisitions provided the most reliable single-site measurements, whereas multi-regional averaging further improved performance, particularly for clinically relevant steatosis thresholds. Although, cohort-derived UGAP thresholds showed promising diagnostic performance; however, these values should be considered exploratory and require validation in larger, multicenter studies before routine clinical implementation. Given the broad availability of ultrasound systems, lower cost, and favorable workflow integration, UGAP represents a practical noninvasive option for liver fat quantification. Future studies should focus on multicenter validation, reproducibility, harmonization of thresholds across populations, and the standardization of acquisition protocols.

## Figures and Tables

**Table 1:** Demographic, clinical, and imaging characteristics of the study population.

Variable	Value
Sample (*n*)	64
Age in years (mean, SD)	54.6 (15.4)
Male sex, *n* (%)	40 (62.5)
BMI, kg/m^2^ (mean, SD)	32.4 (5.7)
Waist circumference, cm (mean, SD)	113.5 (14.6)
Type 2 Diabetes, *n* (%)	36 (56.2)
Hypertension, *n* (%)	34 (53.0)
Obesity, *n* (%)	34 (53.0)
MRI-PDFF, % (mean, SD)	10.1 (7.9)
UGAP right lobe, dB/cm/MHz (median, IQR)	0.70 [0.62–0.80]

Data are presented as mean (standard deviation), median [interquartile range], or number (%), as appropriate. BMI, body mass index; MRI-PDFF, magnetic resonance imaging proton density fat fraction; UGAP, ultrasound-guided attenuation parameter.

**Table 2:** Regional correlations between UGAP and segment-matched MRI-PDFF measurements.

UGAP Region	MRI-PDFF	r (Spearman’s)	*p*-Value
Right center (V, VIII)	mean (V + VIII)	0.91	<0.001
Right caudal (V, VI)	mean (V + VI)	0.86	<0.001
Right cephalic (VII, VIII)	mean (VII + VIII)	0.89	<0.001
Left (II, III)	mean (II + III)	0.74	<0.001

Spearman correlation coefficients (r) describe the association between UGAP values obtained from different hepatic regions and anatomically matched MRI-PDFF reference measurements. Segmental MRI-PDFF values were averaged according to the corresponding UGAP acquisition region.

**Table 3:** Diagnostic performance of single-region and multi-regional UGAP for detection of hepatic steatosis using MRI-PDFF thresholds.

MRI-PDFF	Region	AUC (95%, Confidence Interval)	Cut-Off	Sensitivity (%)	Specificity (%)
≥S1 (≥5.75%)	Right center	0.86 (0.75–0.95)	0.67	85.0	83.0
	Mean 4-region UGAP	0.86 (0.77–0.94)	0.69	80.0	83.0
≥S2 (≥15.5%)	Right center	0.96 (0.91–0.99)	0.79	100.0	86.0
	Mean 4-region UGAP	0.97 (0.92–1.00)	0.79	93.0	96.0
≥S3 (≥21.35%)	Right center	0.96 (0.91–1.00)	0.80	100.0	82.0
	Mean 4-region UGAP	0.98 (0.94–1.00)	0.80	100.0	93.0
S2/S3 (≥15.5%)	Right center	0.96 (0.91–0.99)	0.79	100.0	86.0

Receiver operating characteristic curve analysis of UGAP for the detection of hepatic steatosis defined according to MRI-PDFF thresholds corresponding to mild (S ≥ 1), moderate (S ≥ 2), and severe (S ≥ 3) steatosis. Multi-regional UGAP represents the mean value of four hepatic acquisition regions. AUC, area under the curve.

**Table 4:** Comparison of manufacturer-recommended and cohort-derived UGAP thresholds for the detection of hepatic steatosis.

Steatosis Stage	Source of Thresholds	Cut-Offs (dB/cm/MHz)	Sensitivity (%)	Specificity (%)	PPV (%)	NPV (%)	Accuracy (%)
S1	Manufacturer [20]	0.65	86.0	61.9	82.2	68.4	78.1
S1	Present Study	0.67	81.4	85.7	92.1	69.2	82.8
S2	Manufacturer [20]	0.71	95.7	75.6	68.8	96.9	82.8
S2	Present Study	0.79	78.3	92.7	85.7	88.4	87.5
S3	Manufacturer [20]	0.77	100.0	80.8	54.5	100.0	84.4
S3	Present Study	0.80	91.7	84.6	57.9	97.8	85.9

**Table 5:** Multivariable linear regression analysis of factors associated with UGAP measurements.

Variable	β Coefficient	95% Confidence Interval	*p*-Value
Age	0.0004	−0.0008 to 0.0016	0.502
BMI	0.0029	−0.0008 to 0.0066	0.123
Waist circumference	0.0008	−0.0007 to 0.0023	0.281
AST	−0.0004	−0.0021 to 0.0012	0.623
ALT	0.0001	−0.0009 to 0.0012	0.788
GGT	−0.0002	−0.0006 to 0.0003	0.492
Total cholesterol	0.0004	−0.0002 to 0.0009	0.182
MRI-PDFF (%)	0.0109	0.0082 to 0.0137	<0.001

## Data Availability

The original contributions presented in this study are included in the article. Further inquiries can be directed to the corresponding author.
